
JOURNAL INFORMATION
==============================
NLM Title Abbreviation: Wirtschaftsdienst
Journal ID: Wirtschaftsdienst
NLM Journal ID: 101086612

Wirtschaftsdienst (Hamburg, Germany : 1949)

ISSN: 0043-6275

ARTICLE INFORMATION
==============================
PMCID: PMC8383016
PMID: 34456381
DOI: 10.1007/s10273-021-2969-3
Article ID: 2969
Article version: 1
Subjects: Kurz Kommentiert

COVID-19-Impfung: Wer nicht impfen will, soll zahlen

COVID-19 vaccination: Those who do not want to vaccinate should pay

Grözinger Gerd groezing@uni-flensburg.de

grid.449681.6 0000 0001 2111 1904 Sozial- und Bildungsökonomik, Europa-Universität Flensburg, Auf dem Campus 1, 24943 Flensburg, Deutschland
Electronic publication date: 2021 Aug 24
Print publication date: 2021
Volume: 101
Issue: 8
First page: 580
Last page: 580
Copyright: © Der/die Autor:in(nen) 2021
License: Open Access: Dieser Artikel wird unter der Creative Commons Namensnennung 4.0 International Lizenz veröffentlicht (https://creativecommons.org/licenses/by/4.0/deed.de).
Open Access wird durch die ZBW — Leibniz-Informationszentrum Wirtschaft gefördert.
License URL: https://creativecommons.org/licenses/by/4.0/

issue-copyright-statement: © The Author(s) 2021

==============================

Die Bundesregierung hat erklärt, dass sie eine Impfflicht bei COVID-19 ablehne. Und Verfassungsrechtler:innen argumentieren, es sei immerhin ein körperlicher Eingriff und manche Langfristfolge auch noch unbekannt (Boehme-Neßler, 2021). Deshalb dürfe dergleichen nur in Ausnahmefällen eingeführt werden. Seit kurzem wird in Deutschland eine solche Ausnahme bei den Masern gesehen, wo ab 2020 Kita-Personal, Lehrer:innen etc. eine Impfung dagegen nachweisen müssen. Wie zu erwarten, landete das Gesetz beim BVerfG, das darüber noch nicht entschieden hat.

Gewarnt wird auch vor einem indirekten Zwang, etwa wenn in Zukunft nur Geimpften besondere Privilegien zukämen. Aktuell sind aber in aller Regel eine COVID-19-Impfung und ein negativer (Schnell-)Test gleichgestellt. Es gibt also eine Alternative. Der Test ist sogar kostenlos, eine nicht geringe Ausgabe des dafür aufkommenden Bundes. Je nach Inanspruchnahme und mittlerweile abgesenkter Vergütung können da leicht einige 100 Mio. Euro pro Monat zusammenkommen (Bund der Steuerzahler, 2021). Immerhin beginnt eine Diskussion darüber, ob nicht dies im Sommer geändert werden sollte, weil und wenn dann tatsächlich allen Erwachsenen ein Impfangebot gemacht werden kann.

Mir erscheint das zu zaghaft. Im Prinzip diskutiert die Ökonomie die dahinterliegenden Problemlagen seit langem unter zwei gut etablierten Rubriken, der Kosten-Nutzen-Rechnung und der negativen externen Effekte. Zum ersten Ansatz: jede Impfung hat eine Soll- und Haben-Seite, soll sagen, es gibt nicht nur den angestrebten Schutzfaktor, sondern auch die Gefahr von Nebenwirkungen. Diese beiden Seiten zu quantifizieren, ist Aufgabe dafür kompetenter wissenschaftlicher Stellen. In Deutschland ist dies die Ständige Impfkommission, kurz STIKO. Sie hat auf Grundlage entsprechender Studien aktuell entschieden, mit Ausnahme einiger klar definierter Gruppen, dass Erwachsenen eine Impfung eindeutig angeraten wird, älteren Kindern und Jugendlichen dagegen nur in bestimmten Sonderfällen (RKI, 2021).

Der Hintergrund der Impfempfehlung für Erwachsene ist die vergleichsweise geringe Häufigkeit schlimmer Nebenwirkungen im Verhältnis sowohl zu der schwererer Krankheitsverläufe bei einer Infektion wie auch der zunehmenden Erkenntnisse über erhebliche Nachwirkungen, das sogenannte Long COVID. Da befinden wir uns auf gut vertrautem Terrain, wo der Staat in vielen Bereichen eine Schutzaufgabe gegenüber zu risikobereiten Bürger:innen wahrnimmt und dies auch bei Nichtbefolgen mit Sanktionen bewehrt. Sowohl die Anschnallpflicht im Auto als auch das weite Feld der Sozialversicherungspflichtigkeit sind Beispiele.

Liegt hier der Hauptaspekt auf dem Schutz für die Akteure selbst, sind die Überlegungen zu negativen Externalitäten auf Dritte gerichtet. COVID-19 ist eine hochansteckende Krankheit und wer sich nicht impfen lässt, gefährdet andere. Die Zahl dieser anderen ist nicht gering anzusetzen. Dazu zählen die jüngeren Generationen, aber auch Erwachsene, die aus medizinischen Gründen nicht geimpft werden sollen, wie Schwangere, und schließlich jene, die trotz Impfung keine ausreichende Anzahl Antikörper entwickeln. Gerade die letztere Gruppe rückt in letzter Zeit verstärkt in den Fokus. Das sind Ältere, Immunsupprimierte (z. B. nach einer Organtransplantation), oder Krebspatienten mit einer Chemotherapie (Tagesschau, 2021).

Negative Externalitäten lassen sich auf der einen Seite durch Ver- und Gebote minimieren, aber auf der anderen auch durch einen verhaltensbeeinflussenden Preis. Ist das erstere Vorgehen aus politischen und/oder verfassungsrechtlichen Gründen problematisch, ist aber der zweite Weg gangbar. Man könnte die Krankenversicherungsbeiträge nach geimpft/nicht-geimpft differenzieren. Wer sich der Impfung ohne nachgewiesen guter Begründung verweigert, zahlt z. B. 50 Euro im Monat mehr. Und ist eine weitere erwachsene Person mitversichert, erhöht sich das bei doppelter Absage entsprechend auf 100 Euro. Die Arbeitgeber blieben außen vor, sie haben hier keinerlei Einflussmöglichkeiten. Und die Kassen wissen über ihre Abrechnungen auch, wenn medizinische Gründe gegen eine Impfung sprechen. Ich bin mir sicher: die Exportweltmeisterschaft ist vielleicht perdu, aber Impfweltmeister können wir immer noch werden!


Literatur

Boehme-Neßler, V. (2021), Es muss und kann ohne Impfpflicht gehen, ZEIT Online, 18. Juli, https://www.zeit.de/politik/deutschland/2021-07/corona-impfung-pflicht-verfassung-aufklaerung.
Bund der Steuerzahler (2021), Was kosten die Corona-Schnelltests die Steuerzahler?, https://www.steuerzahler.de/aktuelles/detail/was-kosten-die-corona-schnelltests-die-steuerzahler/.
RKI (2021), STIKO-Empfehlungen zur COVID-19-Impfung, Epidemiologisches Bulletin, 27/2021, https://www.rki.de/DE/Content/Infekt/Impfen/ImpfungenAZ/COVID-19/Impfempfehlung-Zusfassung.html.
Tagesschau (2021), Warum nicht alle Antikörper bilden, 17. Juni, https://www.tagesschau.de/inland/gesellschaft/immunitaet-109.html.
